# Role of *Per3*, a circadian clock gene, in embryonic development of mouse cerebral cortex

**DOI:** 10.1038/s41598-019-42390-9

**Published:** 2019-04-10

**Authors:** Mariko Noda, Ikuko Iwamoto, Hidenori Tabata, Takanori Yamagata, Hidenori Ito, Koh-ichi Nagata

**Affiliations:** 1 Department of Molecular Neurobiology, Institute for Developmental Research, Aichi Human Service Center, Kasugai, Japan; 2 Department of Pediatrics, Jichi medical university, Tochigi, Japan; 3 Department of Neurochemistry, Nagoya University Graduate School of Medicine, Nagoya, Japan

## Abstract

Per3 is one of the primary components of circadian clock system. While circadian dysregulation is known to be involved in the pathogenesis of several neuropsychiatric diseases. It remains largely unknown whether they participate in embryonic brain development. Here, we examined the role of clock gene *Per3* in the development of mouse cerebral cortex. *In situ* hybridization analysis revealed that *Per3* is expressed in the developing mouse cortex. Acute knockdown of *Per3* with *in utero* electroporation caused abnormal positioning of cortical neurons, which was rescued by RNAi-resistant Per3. Per3-deficient cells showed abnormal migration phenotypes, impaired axon extension and dendritic arbor formation. Taken together, Per3 was found to play a pivotal role in corticogenesis via regulation of excitatory neuron migration and synaptic network formation.

## Introduction

The circadian rhythm is self-sustained oscillations in physiology and behavior with endogenous periods of approximately 24 h, which is controlled by a system of positive and negative feedback loops of clock genes with rhythmic expression patterns in a day^1^. Period gene (*Per*) is the first identified circadian clock component in Drosophila^2^. Mammalian Per family has three homologs (Per1, 2, and 3). Circadian clocks are present in the brain and peripheral tissues. The rhythm is coordinated by a master clock in the suprachiasmatic nucleus (SCN), the primary circadian pacemaker in the hypothalamus of mammalian brain^3,4^. Per3 is not only expressed in the SCN^5^, but also distributed throughout the brain in human and adult mouse^6–8^. In mammals, the autonomous rhythm of an individual cell is entrained by hormonal and neuronal signals from the master clock^9^. While the circadian rhythm can be synchronized to environmental cues such as the light-dark cycle and food availability, it controls various physiological processes such as sleep and wakefulness, digestion, body temperature, heart activity, hormone secretion and blood pressure^4,10,11^. In addition, circadian clock genes regulates the onset of postnatal brain development in the mouse neocortex^12^. A variety of health problems thus can result from a disturbance of the circadian rhythm^13^, although the function of circadian clock genes in embryonic brain development, particularly outside the SCN, remains poorly understood. The core circadian clock mechanism is composed of 2 interlocked transcriptional negative feedback loops^14^. In the primary loop, transcriptional activators, BMAL1 (ARNTL) and CLOCK (or its ortholog NPAS2), form a DNA-binding heterodimer and drive expression of the *PER1-3* and *CRY1/2* genes, which ultimately repress BMAL1-CLOCK activity in a feedback manner. This loop also drives rhythmic expression of the nuclear hormone receptors, NR1D1 (Rev-erbα) and NR1D2 (Rev-erbβ), which in turn rhythmically repress the expression of BMAL1 and CLOCK as the second loop^15^. Sleep disturbance has been commonly associated with children suffering from neuropsychiatric diseases^16–18^, and persistent sleep disturbance produced neuronal damage and impaired brain development^19–21^. Therefore, circadian-relevant proteins are likely to play pivotal roles in brain development, and their impaired functions are possible to contribute to the etiology and pathophysiology of neuropsychiatric disorders, including autism spectrum disorder (ASD).

In the present study, we examined the role of Per3 in embryonic brain development. Per3 deficiency caused defects in positioning, migration, axon growth, and dendrite development of excitatory neurons during corticogenesis, while it appeared not to be involved in proliferation of neuronal stem and progenitor cells. The results obtained indicate that Per3 plays a crucial role in brain development and its loss-of-function due to haploinsufficiency might contribute to the etiology of neuropsychiatric diseases.

## Results

### Characterization of RNAi vectors and expression plasmids

**Figure 1:**
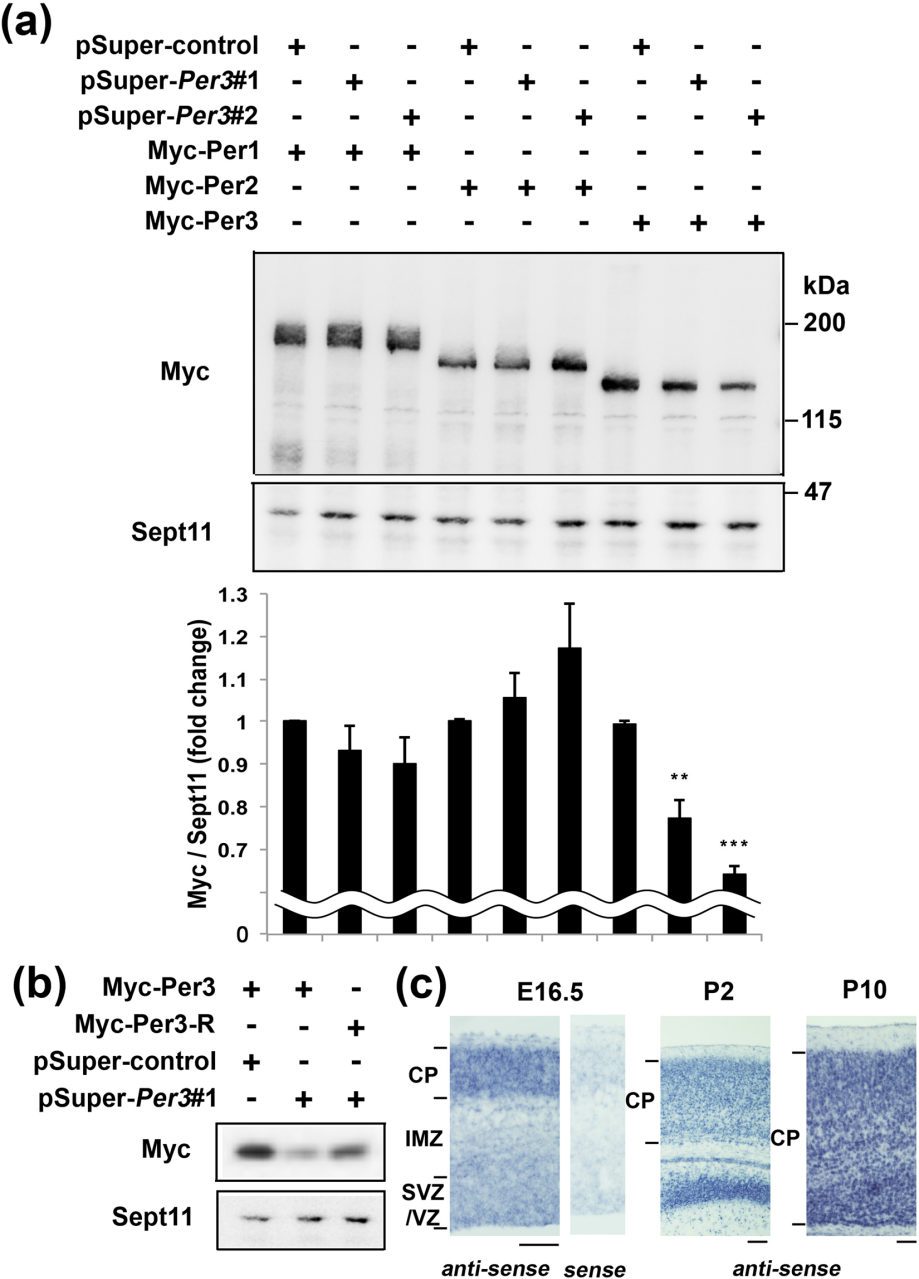
Characterization of RNAi vectors for Per3. (**a**) pCAG-Myc-Per1, –Per2 or –Per3 was co-transfected into COS7 cells with control pSuper vector, pSuper-mPer3#1 and #2 in various combinations. After 48 h, cells were harvested and subjected to western blotting (20 µg protein per lane) with anti-Myc. Anti-Sept11 was used for a loading control. Molecular weight markers are shown at the *right*. Relative levels of Myc-Per proteins were calculated with ImageJ software based on densitometry and normalized against Sept11. Error bars indicate SD (n = 3). The data shown are representatives of 3 independent experiments. **P 0.01 and ***P < 0.001 (control vs RNAi) by one-way ANOVA < with Fisher’s LSD post hoc test. (**b**) Characterization of RNAi-resistant Per3, Per3-R. pCAG-Myc-Per3 or –Per3-R was co-transfected into COS7 cells with pSuper-control or pSuper-mPer3#1. Analyses were done as in (**a**). (**c**) Coronal brain sections prepared from E16.5, P2 and P10 mice were examined by *in situ* hybridization with m*Per3* riboprobe. Note that only weak signal was detected with the sense probe at E16.5. Scale bars, 100 μm.

We designed 2 RNAi vectors, pSuper-mPer3#1 and #2, against distinct regions in the mouse *Per3* coding sequence. While these vectors efficiently knocked down Per3 expression in COS7 cells, they did not affect the expression of Per3-related molecules, Per1 and Per2 (Fig. 1a). We prepared an RNAi-resistant version of Per3, Per3-R, and confirmed its resistance to pSuper-mPer3#1 (Fig. 1b).

### Role of Per3 in excitatory neuron migration during corticogenesis

While clock genes (including *Per1* and *Per2*) were expressed in the developing mouse cortex at P10^12^, expression pattern of *Per3* has not been fully elucidated. We thus performed *in situ* hybridization (ISH) to determine the *Per3*-mRNA expression in the developing mouse brain. As shown in Fig. 1c, cells in the ventricular zone (VZ)/subventricular zone (SVZ) and the cortical plate (CP) expressed significant amount of *Per3* at E16.5. At this stage, CP cells exhibited higher expression compared to the VZ/SVZ cells. Expression of *Per3*-mRNA gradually increased during postnatal development (Fig. 1c). We also tried to clarify the expression pattern of Per3 protein by immunohistochemical analysis. However, both commercial and homemade antibodies could not detect endogenous Per3 protein under our experimental conditions (data not shown).

**Figure 2:**
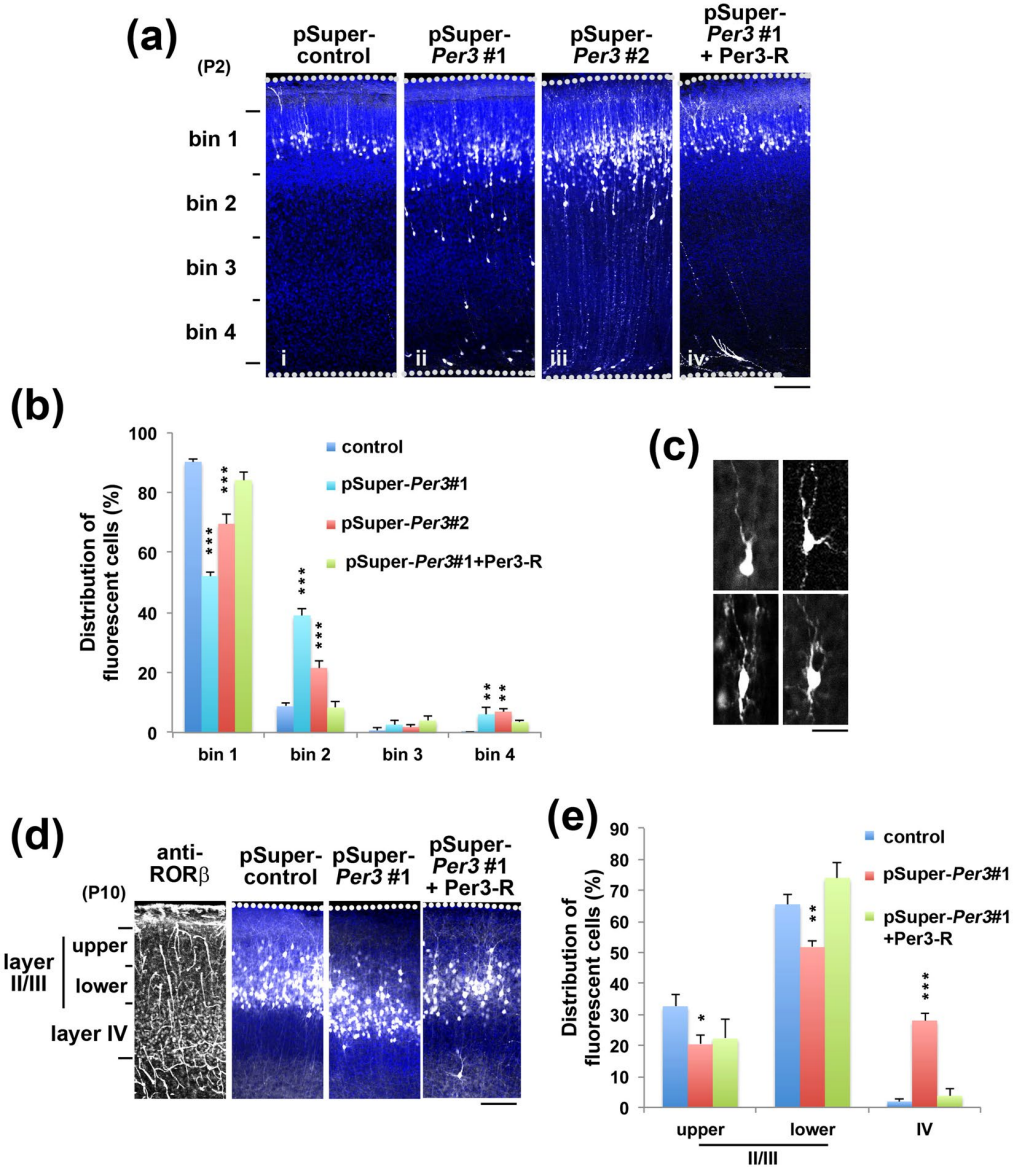
Effects of Per3-knockdown on neuronal migration during corticogenesis. (**a**) pCAG-EGFP was co-electroporated with control pSuper (i), pSuper-mPer3#1 (ii) or #2 (iii) into cerebral cortices at E14.5. For the rescue experiments, pCAG-EGFP was co-electroporated with pSuper-mPer3#1 together with pCAG-Myc-mPer3-R (iv). Coronal sections were prepared at P2. Nuclei were stained with DAPI (blue). Dotted lines represent the pial and ventricular surfaces. Scale bars in (**a,d**), 100 µm. (**b**) Quantification of the distribution of GFP-positive neurons in distinct regions of cerebral cortex for each condition shown in (**a**). Error bars indicate SD; control (n = 5), pSuper-mPer3#1 (n = 5), pSuper-mPer3#2 (n = 3), pSuper-mPer3#1 + Per3-R (n = 5). *P < 0.05, **P < 0.01 and ***P < 0.001 (vs. control) by one-way ANOVA with Fisher’s LSD *post hoc* test. (**c**) Representative images of Per3-deficient neurons migrating in the lower CP. Scale bar, 10 µm. (**d**) Migration defects of Per3-deficient cortical neurons at P10. Coronal sections were immunostained with anti-RORβ as a layer IV marker. Dotted lines represent the pial surface. Analyses were done as in (**a**). Note that vascular structure was nonspecifically stained. (**e**) Quantification of the distribution of GFP-positive neurons in distinct regions in (**d**). Error bars indicate SD; control (n = 9), pSuper-mPer3#1 (n = 8), pSuper-mPer3#1 + Per3-R (n = 3). *P < 0.05, **P < 0.01 and ***P < 0.001 (vs. control) by Fisher’s LSD.

From the results of ISH, we considered that functional defects in Per3 could induce abnormal brain development which might be related to the etiology of neuropsychiatric diseases. Thus, we examined the effect of acute *Per3*-silencing on the migration of newly generated cortical neurons by *in utero* electroporation. The progenitor and stem cells in the VZ of E14.5 mice brains were transfected with pCAG-GFP in the presence of pSuper-control, pSuper-mPer3#1 or #2. When the localization of transfected cells and their progeny was analyzed at P2, control neurons migrated normally to the superficial layer (bin1; layer II-III) of the CP (Fig. 2a,i and b). In contrast, cells transfected with pSuper-mPer3#1 or #2 frequently remained in the lower part of the CP (Fig. 2a,ii and iii and b). One-way ANOVA revealed significant effects of the RNAi vectors in bin1 (*F*_*5,20*_ = 95.204, *P* < 0.0001); bin2 (*F*_*5,20*_ = 73.258, *P* < 0.0001); bin3 (*F*_*5,20*_ = 77.368, *P* = 0.1491) and bin4 (*F*_*5,20*_ = 8.258, *P* = 0.0025). *Post-hoc* tests revealed abnormal migration in neurons transfected with pSuper-mPer3#1 or #2 vectors compared to the control (Fig. 2b). Rescue experiments were then conducted to rule out off-target effects. When pCAG-EGFP was electroporated into the VZ cells with pSuper-mPer3#1 together with pCAG-Myc-Per3-R, the positional defects were significantly rescued at P2 (Fig. 2a,iv and b). Based on these results, we used pSuper-mPer3#1 in the subsequent analyses. When the morphology of Per3-deficient migrating neurons was examined in the lower CP, abnormal multipolar-shaped cells were frequently observed (Fig. 2c).

When we examined the long-term effects of *Per3*-knockdown, the deficient cells could not completely make it to the layer II/III and many of them were still abnormally positioned at the RORβ-positive layer IV at P10 (Fig. 2d,e). We consider that Per3-deficiency prevented, rather than delayed, cortical neuron migration. One-way ANOVA revealed significant effects of the RNAi vectors on the localization of cells in bin1 (*F*_*2,17*_ = 3.546, *P* = 0.052), bin2 (*F*_*2,17*_ = 10.340, *P* = 0.001) and bin3 (*F*_*2,17*_ = 77.350, *P* < 0.0001).

Taken together, loss-of-function of Per3 appeared to prevent cortical neuron migration, leading to abnormal cortical architecture.

### Per3 is not involved in the cell cycle of progenitor and stem cells in the VZ

Since neuronal migration delay was observed when cell cycle is prolonged^22^, we looked into the possible role of Per3 in the cell cycle regulation of neuronal progenitor and stem cells in the VZ. When the impact of *Per3*-knockdown on the cell cycle was examined by labeling S-phase cells with EdU to detect DNA replication, Per3-deficient cells entered S-phase to a similar extent as the control cells and G1-progression rate did not statistically differ between control and the deficient cells (Supplementary Fig. 1a,b). We then examined the effect of *Per3*-knockdown on the cell cycle re-entry by staining EdU and Ki67, a marker for all active phases of the cell cycle except the quiescent G0 state. In this analysis, EdU/Ki67-double positive cells represent cells still proliferating after EdU incorporation, while EdU-positive but Ki67-negative cells show differentiated (no longer proliferating) neurons. Per3-deficient cells tended to re-enter the cell cycle and there is no statistical difference from the control cells (Supplementary Fig. 1c,d). Collectively, we concluded that Per3-deficiency does not influence the cell cycle in VZ cells, and that abnormal neuron positioning by the knockdown was attributable to cell migration defects.

### Time-lapse imaging of migration of Per3-deficient neurons

**Figure 3:**
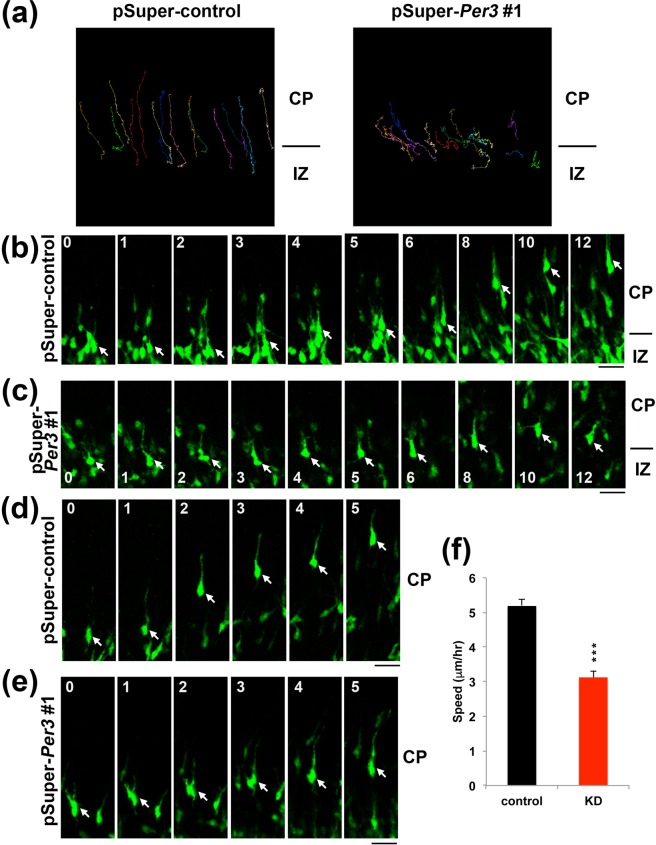
Time-lapse imaging of migration of Per3-deficient neurons. Analyses were repeated 3 times for each case, and the migration pattern was observed for 20 cells in each imaging. Representative results were shown in (**a–e**). (**a**) Tracing of control and Per3-deficient cells in the IZ-CP boundary. E14.5 cortices were co-electroporated with pCAG-EGFP together with control pSuper or pSuper-mPer3#1, followed by coronal section slice preparation at E16.5 and time-lapse imaging for 20 h. Scale bars in (**b–e**), 20 µm. (**b,c**) Migratory tracks of control (**b**) and Per3-deficient neurons (**c**) in the IZ/CP boundary. (**d,e**) Migratory tracks of control (**d**) and the deficient neurons (**e**) in the lower-middle CP. (**f**) Calculation of migration velocity of control and the deficient cells in the middle-upper CP. More than 10 cells were analyzed in each experiment (*n* = 3). Error bars indicate SD. ***P < 0.0001 by Students *t*-test.

The abnormal positioning of Per3-deficient cortical neurons may be due to reduced migration velocity. Alternatively, the positional defects may be attributable to migration into aberrant directions in the CP and/or a positioning program error based on disrupted gene expression leading to dysfunction of proteins crucial for cellular processes such as morphology and adhesion. To address this issue, we performed time-lapse imaging of Per3-deficient neurons migrating in the intermediate zone (IZ) and CP. To this end, VZ cells were electroporated *in utero* with pCAG-EGFP together with control pSuper or pSuper-mPer3#1 at E14.5, and migration was monitored from E16.5 for ~20 h. At the beginning of imaging, Per3-deficient neurons displayed localization and multipolar morphology similar to those of the control cells (data not shown). In the control experiment, GFP-positive neurons normally transformed from multipolar to bipolar in the upper IZ and moved into the CP (Fig. 3a *left* panel, b, and Supplementary Video 1). On the contrary, Per3-deficient cells tended to be stranded at the IZ/CP boundary and many of them did not transform into bipolar status efficiently (Fig. 3a, *right* panel, c, and Supplementary Video 2).

When time-lapse imaging was continued further to monitor migration in the lower – middle CP, control cells showed typical radial migration (Fig. 3d, Supplementary Video 3). On the other hand, while many of the deficient cells remained multipolar, considerable portion of them showed apparently normal radial migration with normal cell morphology in the CP (Fig. 3e, Supplementary Video 4). Their average migration velocity, however, was lower compared with the control neurons (Fig. 3f). We suppose that incomplete Per3-depletion in cortical neurons incorporating low amount of the RNAi vector limitedly affected the migration mode.

### Involvement of Per3 in the axon elongation *in vitro* and *in vivo*

**Figure 4:**
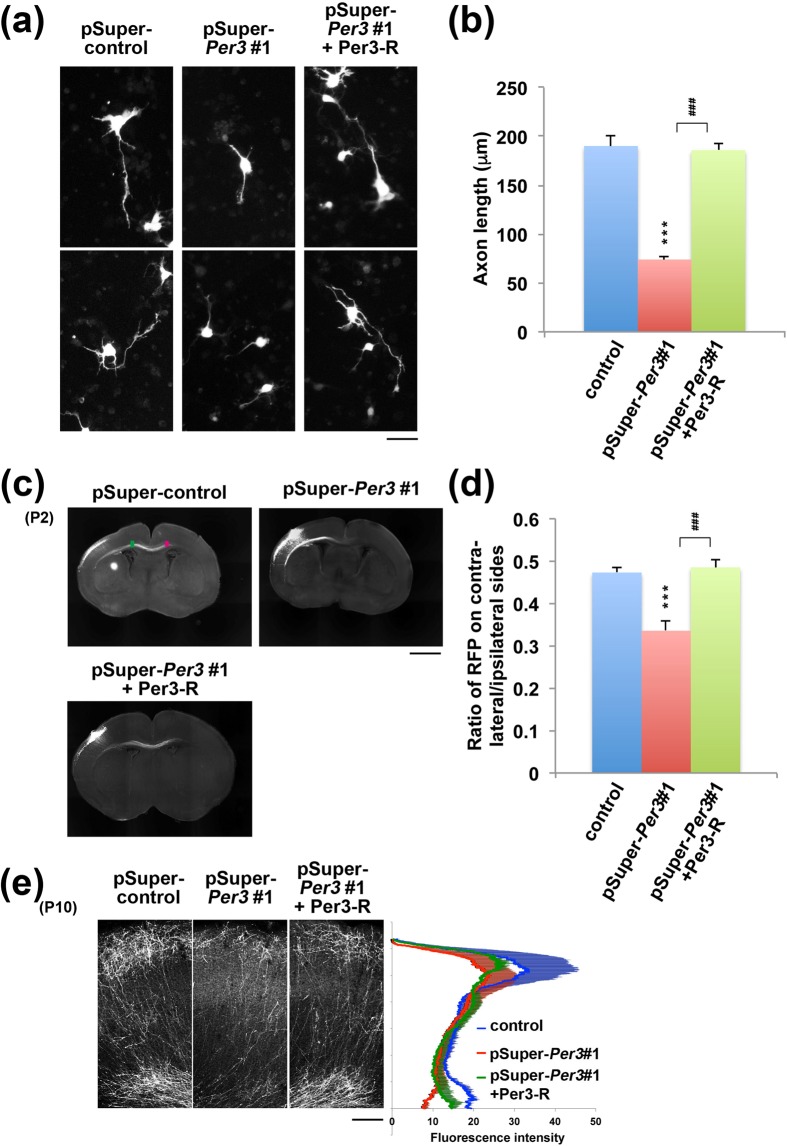
Effects of Per3-knockdown on axon growth. (**a**) Cortical neurons isolated at E14.5 were co-transfected with pCAG-EGFP together with control pSuper, pSuper-mPer3#1 or pSuper-mPer3#1+pCAG-Myc-Per3-R. At day 3 *in vitro*, cells were fixed and stained for GFP. Scale bar, 10 µm. (**b**) Quantitative analysis of axon growth in (**a**). Axon was recognized as the longest neurite. Axonal length was quantified using ImageJ software. Error bars indicate SD (n = 3). ***P < 0.0001 and ^###^*P* < 0.0001 by Fisher’s LSD. (**c**) pCAG-Turbo-RFP was co-electroporated *in utero* with control pSuper, pSuper-mPer3#1 or pSuper-mPer3#1+pCAG-Myc-Per3-R into cerebral cortices at E14.5. Coronal sections were prepared at P2 and stained for RFP. Scale bar, 500 µm. (**d**) Quantitative analyses of axon growth in (**c**). The ratio of the intensity of RFP-positive axon in the area (*green*) of electroporated ipsilateral cortex to that in the area (*magenta*) of contralateral cortex in (**c**) was calculated with ImageJ software. Error bars indicate SD (n = 3). ***P < 0.0001 and ^###^*P* < 0.0001 by Fisher’s LSD. (**e**) Estimation of axon growth at P10. Representative images of the terminal arbors of axons expressing RFP with control pSuper, pSuper-Per3#1 or pSuper-Per3#1+Per3-R were shown. The experiments were repeated 3 times. Densitometric analyses of RFP fluorescence intensity were shown as blue (control), red (pSuper-mPer3#1) and green (pSuper-mPer3#1+Per3-R) lines. Shadows represent SD (control, n = 3; pSuper-mPer3#1, n = 3; pSuper-mPer3#1+Per3-R, n = 3). Scale bar, 200 µm.

Since functional defects in Per3 are possible to disrupt axon elongation during corticogenesis, we analyzed the effects of *Per3*-knockdown on neurite elongation of primary cultured mouse cortical neurons. At days *in vitro* (DIV) 3, axon length was significantly shorter in *Per3*-deficient neurons (Fig. 4a,b). Notably, this phenotype was rescued by RNAi-resistant Per3-R (Fig. 4a,b).

We further explored *in vivo* the effects of *Per3*-knockdown on the axon elongation. When *Per3* was silenced in the VZ cells at E14.5 and axon elongation was observed at P2, axon density of the deficient neurons became lower after crossing the corpus callosum (CC) when compared to the control cells (Fig. 4c,d). This phenotype was rescued by Per3-R (Fig. 4c,d). When we tested whether axons of the deficient neurons eventually ended up extending normally, the elongation was still delayed slightly but significantly at P10 (Fig. 4e). It is notably that the phenotype was at least partially rescued by Per3-R (Fig. 4e).

Taken together, Per3 appeared to be involved in axon growth of excitatory neurons from the ipsilateral to the contralateral cortex. Disturbance of Per3 function might affect synapse network formation.

### Per3 is involved in the regulation of dendritic arbor formation *in vivo*

**Figure 5:**
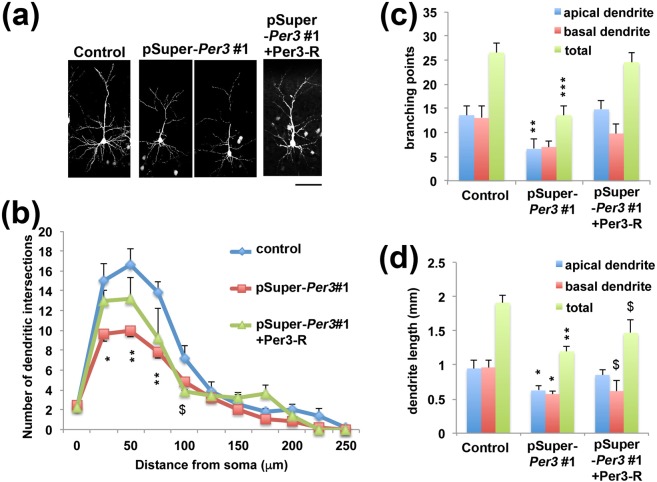
Effects of Per3-knockdown on dendritic growth. (**a**) pCAG-loxP-GFP was co-electroporated for sparse expression with pCAG-M-Cre together with pSuper-control, pSuper-mPer3#1 or pSuper-mPer3#1+pCAG-Myc-Per3-R into cerebral cortices at E14.5. Analyses were carried out with cortical slices at P10. Representative average Z-stack projection images of GFP fluorescence of cortical neurons in the upper CP were shown. Scale bar, 50 µm. (**b**–**d**) One section from each brain was analyzed for pSuper-control (*n* = 5), pSuper-Per3#1 (*n* = 7) and pSuper-mPer3#1 + Per3-R (n = 5). (**b**) Branch point number of dendrites was analyzed by Sholl test. Error bars indicate SD (n = 3). Branching point number (**c**) or total length (**d**) was calculated for apical as well as basal dendrites. *P < 0.05, ^$^*P* < 0.05, **P < 0.01 and ***P < 0.001 (*control vs pSuper-Per3#1, ^$^control vs pSuper-Per3#1+Per3-R) by Fisher’s LSD.

Since dendritic arbor formation is essential for synaptic network formation, we examined the role of Per3 in dendrite development. Introduction of pSuper-mPer3#1 at E14.5 into VZ cells resulted in highly abrogated dendritic arborization at P10, indicating that Per3 plays an essential role in dendritic growth (Fig. 5a). It should be noted that we examined dendrite growth using normally positioned cortical neurons. Many Per3-deficient neurons reached the superficial layer of cerebral cortex perhaps due to the incomplete *Per3*-silencing by incorporating low amount of the RNAi vector (Fig. 2a). We consider that dendritic development is more sensitive to the Per3 expression level than neuronal migration. Sholl analysis revealed that the deficient cells had decreased branch point number compared to the control cells (Fig. 5b). These abnormal phenotypes observed were at least partially rescued by Per3-R (Fig. 5a,b). Reduction of the number was detected for both apical and basal dendrites (Fig. 5c). Total length of apical and basal dendrites also became shorter when *Per3* was knocked down (Fig. 5d). One-way ANOVA revealed significant effects of the RNAi vectors in apical dendritic length (*F*_*2,12*_ = 3.372, *P* = 0.069), basal dendritic length (*F*_*2,12*_ = 3.863, *P* = 0.051), total dendritic length (*F*_*2,12*_ = 7.586, *P* = 0.007), apical dendritic branches (*F*_*2,12*_ = 8.970, *P* = 0.004), basal dendritic branches (*F*_*2,12*_ = 2.265, *P* = 0.146) and total dendritic branches (*F*_*2,12*_ = 12.374, *P* = 0.001). Expression of Per3-R partially recovered the morphological defects of apical and basal dendrites of layer II/III pyramidal neurons (Fig. 5c,d).

Collectively, the obtained data suggest that Per3 plays a crucial role in dendrite growth of excitatory neurons. Functional loss of Per3 is most likely to impair neuronal connectivity through disturbance of dendrite growth as well as axon elongation.

## Discussion

While sleep is thought to be essential for synaptic development and brain maturation^23^, possible interplay of synaptic and clock genes has been proposed in neuropsychiatric disorders pathogenicity^24,25^. We have identified variations in circadian-related genes, including *PER3* and *NR1D1*, in ASD patients with sleep disorders^26^, and reported the role of an *NR1D1* mutation in the ASD pathogenesis^27^. As for *PER3*, associations between genetic polymorphisms of the gene and mental disorders such as schizophrenia and bipolar disorder have been reported^28–30^. In the present study, we explored the effects of functional defects of Per3 on mouse brain development using *in utero* electroporation technique. At first, we determined the *Per3*-mRNA expression in cerebral cortex during embryonic to postnatal development (Fig. 1c). Then, we examined the effects of RNAi-mediated disruption of *Per3* on neuronal migration in the developing brain. Per3-deficient neurons were distributed significantly at the lower part of cerebral cortex, perhaps due to the delay of radial migration (Fig. 2a,b). It should be noted here that pSuper-mPer3#1 and #2 exhibited different knockdown effects in COS7 cell transient expression experiments and *in utero* electroporation analyses, as in the analyses of Phactr1^31^ and Nr1d1^27^. We assume that the discrepancy was due to the difference of knockdown efficiency between COS7 cells *in vitro* and cortical neurons *in vivo*. Different knockdown efficiency of the RNAi vectors on overexpressed protein and endogenous one also might underlie the discrepancy. In addition to the abnormal positioning, interhemispheric axon projection and dendritic arbor formation were abrogated when *Per3* was silenced in the developing mouse cerebral cortex. Based on the data obtained, we concluded that Per3 plays an essential role in the establishment of cortical architecture.

Since Per3-deficient neurons tended to be stuck at the CP/IZ boundary (Fig. 3a and Supplementary Video 2), this molecule appears to be involved in the regulation of the multipolar - bipolar transition. This transition has been reported to be regulated by GSK3β -mediated signaling^32^. Interestingly, GSK3β regulates the phosphorylation and stabilization of Nr1d1^33,34^, which interacts, albeit indirectly, with Per3 in the feedback loop of Clock components. Although the direct functional interaction between Per3 and GSK3β remains to be clarified, Per3 might be regulated by post-translational modification such as phosphorylation.

The results of neuronal migration analyses suggest that Per3-deficient neurons have defects in cytoskeletal function and/or adhesion to the radial glial fibers. While there are no reports describing direct association of Per3 with cytoskeleton- or cell adhesion-related proteins, Per3 may regulate the expression of these molecules as a component of a transcription machinery. In this context, Per family member Per2 was shown to regulate fibronectin expression which is crucial for tumor cell motility^35^. Since Per3 plays a role in gene expression, disruption of its function may cause defects in not only cell morphology and migration but also yet unidentified function(s) of cortical neurons. On the other hand, CK1δ, a negative feedback loop regulator of Per proteins, was reported to interact with tubulin^36^, suggesting functional interaction of Per3 with microtubules.

As for the functional significance of circadian clock proteins in cortical development, Nr1d1 has been shown to be involved in neuronal architecture and function during brain development, through the interaction with Oligophrenin-1^37^, which is known to regulate dendritic spine morphology^38^. Notably, abnormal behaviors were observed in *Nr1d1-*knockout mice, such as marked hyperactivity, impaired response habituation in novel environments, deficient contextual memories and impairment in nest-building ability^39^. In the meanwhile, *Per3*-knockout mice have revealed only small changes in the light-sensitive behaviors^5,40^. Further intensive analyses with the knockout mice are possible to clarify the role of Per3 in corticogenesis.

Taken together, we here clarified that Per3 plays a pivotal role in corticogenesis via regulation of excitatory neuron migration and synaptic network formation. It remains to be elucidated if the ablation of neuronal network formation and/or maintenance really occurs in patients of neurodevelopmental disorders with *PER3* mutation, and, if so, how the abnormality determines the clinical features. Further intensive analyses are required to address these issues.

## Methods

### Ethics approval

We followed the fundamental guidelines for proper conduct of animal experiments and related activity in academic research institution under the jurisdiction of the Ministry of Education, Culture, Sports, Science and Technology, Japan. All the protocols for animal handling and treatment were reviewed and approved by the animal care and use committee of Institute for Developmental Research, Aichi Human Service Center (approval number M-10). All experiments were conducted in compliance with the ARRIVE guidelines. All methods, data presentation and statistical analyses were carried out in accordance with the ‘TOP’ guidelines. This study has not been pre-registered.

### Plasmids

Mouse *Per1-3* cDNAs were kind gifts from Dr. T. Takumi (RIKEN, Saitama, Japan), and cloned into pCAG-Myc vector (Addgene Inc., Cambridge, MA). For RNAi experiments, following target sequences were inserted into pSuper-puro vector (OligoEngine, Seattle, WA): mPer3#1, CAGTAACGACAAAGACATA (1170-1188); mPer3#2, CCTAGATGCTCTTAACTAT (225-243). Numbers indicate the positions from Per3 translational start sites. We named these vectors as pSuper-mPer3#1 and #2. For the control RNAi experiments, we used pSuper-Luc designed against luciferase (CGTACGCGGAATACTTCGA). To generate RNAi-resistant Per3, Per3-R, silent mutations were introduced, as underlined, in the target sequence (GAGCAATGATAAGGATATC in *Per3*#1). All constructs were verified by DNA sequencing. pCAG-histone 2B-EGFP was used to label chromosomes^41^.

### Primary antibodies

Polyclonal rabbit anti-GFP and anti-Myc antibodies were prepared and characterized previously^42^. Polyclonal rabbit antibody against a cytoskeleton-related protein, Sept11, was prepared as described^43^. Mouse monoclonal anti-Myc (MBL, Nagoya, Japan) and anti-RORβ antibodies (Perseus Proteomics, Tokyo, Japan) were also used.

### Cell culture, transfection, western blotting and immunofluorescence

COS7 cell transfection experiments were performed with Lipofectamine 2000 **(**Life Technologies Carlsbad, CA**)**. Primary cultured mouse cortical neurons were prepared essentially as described^44^. Briefly, cerebral cortices of ICR mouse embryos at E14.0 were microdissected and treated with DNaseI and Papain (Nacalai Tesque, Kyoto, Japan). Then, plasmids were transfected into the primary cortical cells by NEPA21 (NEPAGENE, Chiba, Japan). SDS-PAGE (10% gel) and western blotting were performed as described^45^. For all western blots, uncropped blot data were provided in Supplementary Information. Immunofluorescence analyses were carried out as described^46^. Alexa Fluor 488- or 568-labeled IgG (Life Technologies) was used as a secondary antibody. Fluorescent images were captured with FV-1000 confocal laser microscope (Olympus, Tokyo, Japan).

### *In situ* hybridization

Coronal sections of mouse brain at E16.5, P2 and P10 were probed with a digoxigenin-labeled antisense riboprobe against full length *Per3* as previously described^47^. The sense riboprobe was used as a negative control.

### *In utero* electroporation, quantitative analysis of neuronal migration, and time-lapse imaging

*In utero* electroporation was carried out at E14.5 essentially as described^48,49^. E14.5 ICR mice were purchased from Japan SLC (Hamamatsu, Japan) and housed individually given *ad libitum* access to food and water. Both female and male pups were used. Pups were maintained in a 12:12 light-dark cycle together with their dams before being sacrificed at indicated time points, which were primary endpoints of this study. At least 5 electroporated brains from different dams were used for each experiment. Distribution of GFP-positive neurons chosen randomly in slices was quantified as follows. Pups were anesthetized on ice for 5 to 10 m affording physical relief and brains were fixed overnight in 4% paraformaldehyde phosphate buffer solution. 100 μm (P2 sample) or 150 μm (P10 sample) sections were prepared by vibratome. The coronal sections of cortices containing the labeled cells were classified into 4bins as described^50^. The number of labeled cells (more than 100 *per* sample) of at least 3 slices *per* brain was calculated. Exact numbers for all experiments are provided in the figure legends. During experiments and analyses, the investigators were blinded to experimental group. Brain samples were identified by electroporated side of hemisphere and given numbers, which were announced to the investigator only after finishing analysis. Live-imaging analyses were conducted as described previously^50^.

### Quantitative analysis of axon elongation and dendritic arbor formation

For estimation of axon growth, RFP signal intensity of callosal axons was measured in the marked areas (170 × 300 µm rectangle) on the ipsilateral (before entering the CC; *green* rectangle) and contralateral (after leaving the CC; *magenta* rectangle) sides (Fig. 4c). We then calculated the ratio of the contralateral signal intensity to that in the corresponding ipsilateral side using ImageJ software. To measure the total length and branching point number of dendrite of post migratory (mature) neurons, images of GFP-positive neurons in the layer II-III at P10 brain were acquired by FV-1000 confocal microscopy. ImageJ software was used for the quantitative analyses of dendritic length and Sholl test.

### EdU (5-ethynil-2′-deoxyuridine) incorporation experiment

Cell cycle analysis was performed essentially as described^50^. Briefly, embryos were electroporated *in utero* with pCAG-H2B-EGFP together with control pSuper or pSuper-mPer3#1 at E14.5. GFP and EdU were detected with anti-GFP and Alexa Fluor555 azide (Life Technologies Japan), respectively. When the ratio of EdU/Ki67-double positive cells to EdU-positive ones was determined, EdU injection was done at 19 h after electroporation. Then, the pregnant mice were anesthetized and brains from embryos were fixed after 24 h and subjected to immunostaining for EdU, Ki67 and GFP. Numbers of cells used for each calculation are more than 100.

### Statistical analysis

Results were expressed as means ± SD. When data were obtained from only 2 groups, Student’s *t*-test was used for comparison. For other experiments, the rate of cell scores was initially analyzed using the one-way analysis of variance (ANOVA). Subsequently, Fisher’s least significant difference test (LSD) was applied to absolute values as a *post hoc* test of multiple comparisons. The level of statistical significance was considered to be *p* < 0.05. Statistical analysis was performed using Statview software (SAS Institute, Cary, NC).

## Supplementary information

Supplementary video 1

Supplementary video 2

Supplementary video 3

Supplementary video 4

Supplementary Information

## Data Availability

All relevant data are within the paper and its Supporting Information files.
