# Supplementary material for: Role of Per3, a circadian clock gene, in embryonic development of mouse cerebral cortex
Source: Sci Rep. 2019 Apr 10;9:5874. doi: 10.1038/s41598-019-42390-9 (PMC6458147; doi:10.1038/s41598-019-42390-9)
Supplement: Supplementary file 5 — Supplementary Information [file 41598_2019_42390_MOESM5_ESM.pdf]

Role of Per3, a circadian clock gene, in embryonic development of mouse cerebral cortex

Mariko Noda[1], Ikuko Iwamoto[1], Hidenori Tabata[1], Takanori Yamagata[2], Hidenori Ito[1] and Koh-ichi Nagata[1,3*]

[1]Department of Molecular Neurobiology, Institute for Developmental Research, Aichi Human Service Center, Kasugai
[2]Department of Pediatrics, Jichi medical university, Tochigi
[3]Department of Neurochemistry, Nagoya University Graduate School of Medicine, Nagoya, Japan
*Corresponding author

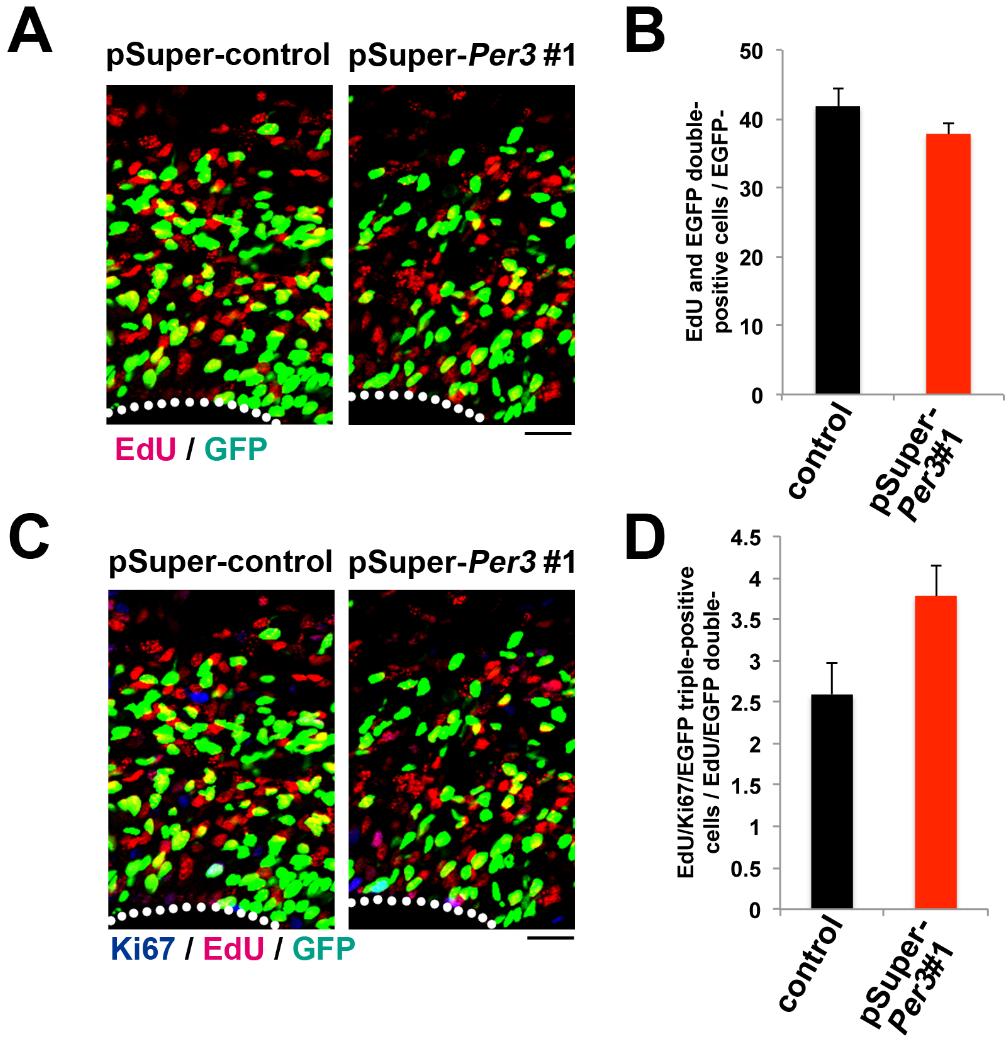

Supplementary Figure 1. Effects of *Per3*-silencing on the cell cycle **(A)** E14.5 cortices were co-electroporated with pCAG-EGFP together with pSuper vector or pSuper-m*Per3*#1. Subsequent procedure was performed as described in "Materials and methods". Coronal sections were immunostained for GFP (green) and EdU (red). Dotted lines represent ventricular surface. Scale bar, 10 µm. **(B)** Quantification of EdU/GFP double-positive cells relative to GFP-positive ones in (A). Error bars indicated SD (n=3). P = 0.2287 by Students t-test. **(C)** Effect of *Per3*-silencing on cell cycle exit. Differentiated neurons are EdU/GFP double-positive (*yellow*) while EdU/Ki67/GFP triple-positive cells (*white*) maintain progenitor potency. Scale bar, 10 µm. **(D)** Quantification of EdU/Ki67/GFP-triple positive cells relative to EdU/GFP-double positive cells in (C). Error bars indicate SD (n=3). P = 0.0504 by Students t-test.

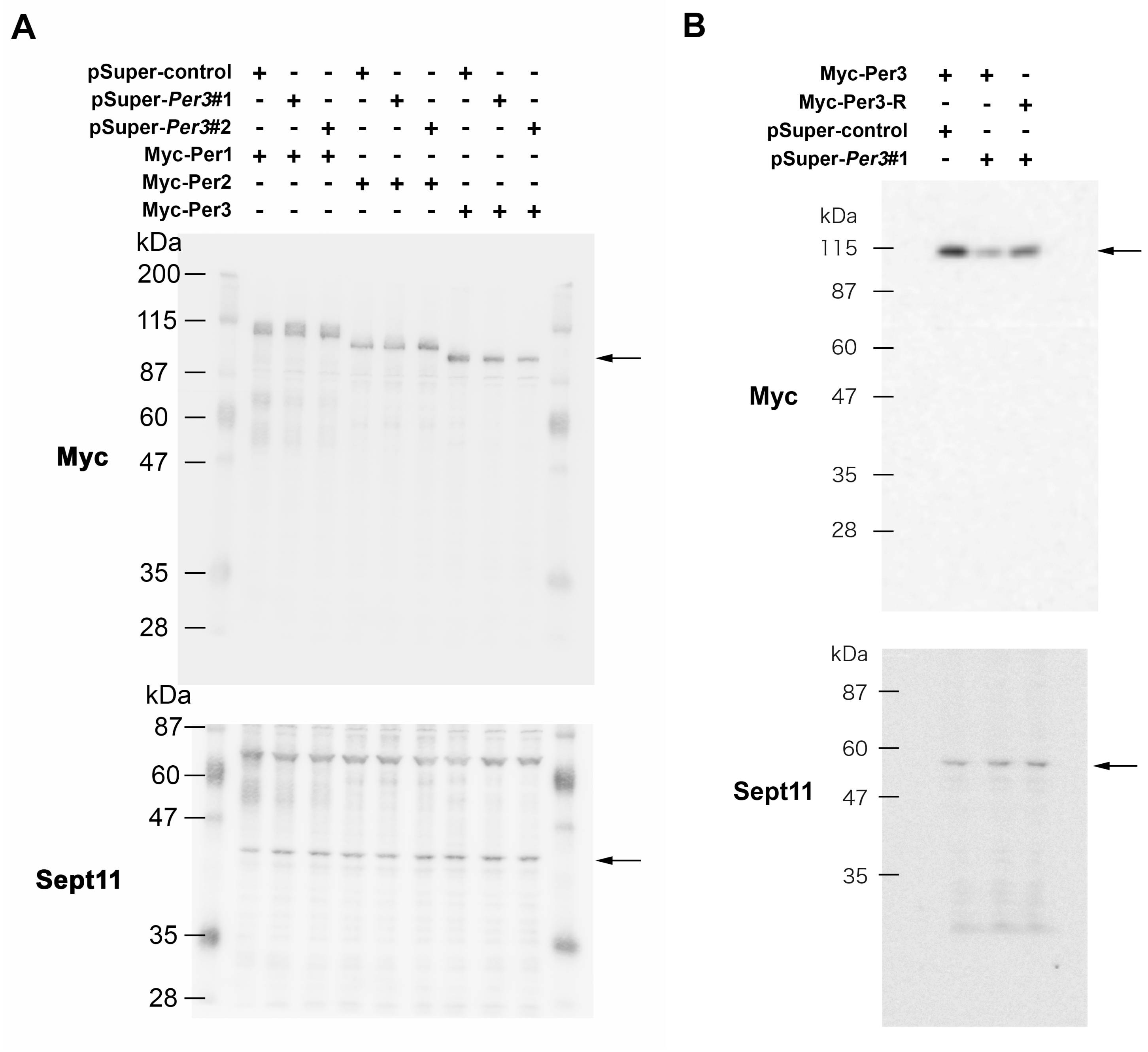

Supplementary Figure 2. Uncropped blots of Figure 1A Western blot of protein samples immunoblotted with anti-Myc and anti-Sept11. The protein bands shown in Figure1 are indicated by arrows.

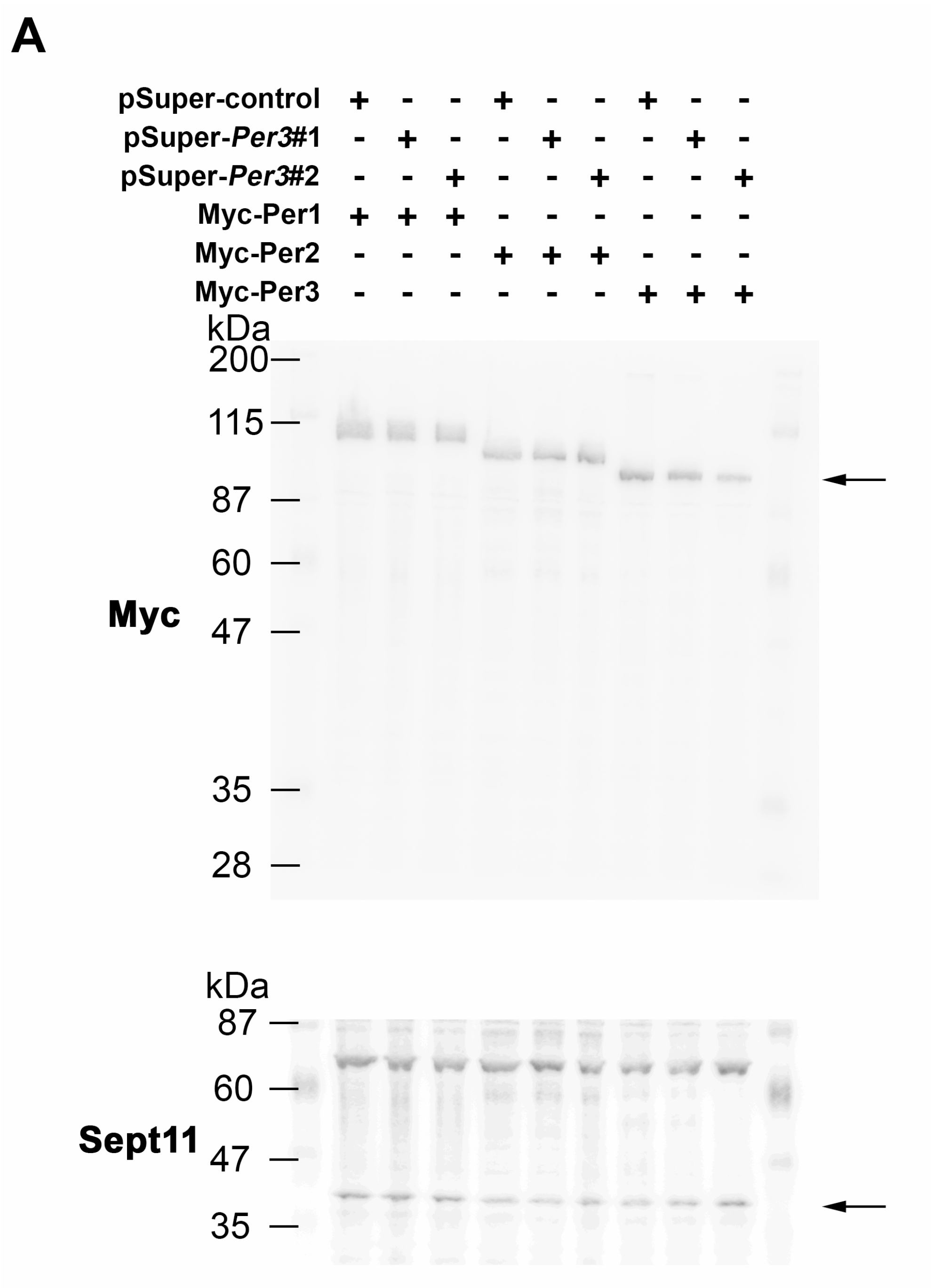

Supplementary Figure 4. Full length blot for densitometry analysis of Figure 1A, as second unrepresented sample Western blot of protein samples immunoblotted with anti-Myc and anti-Sept11. The protein bands used to the analysis in Figure1A are indicated by arrows.

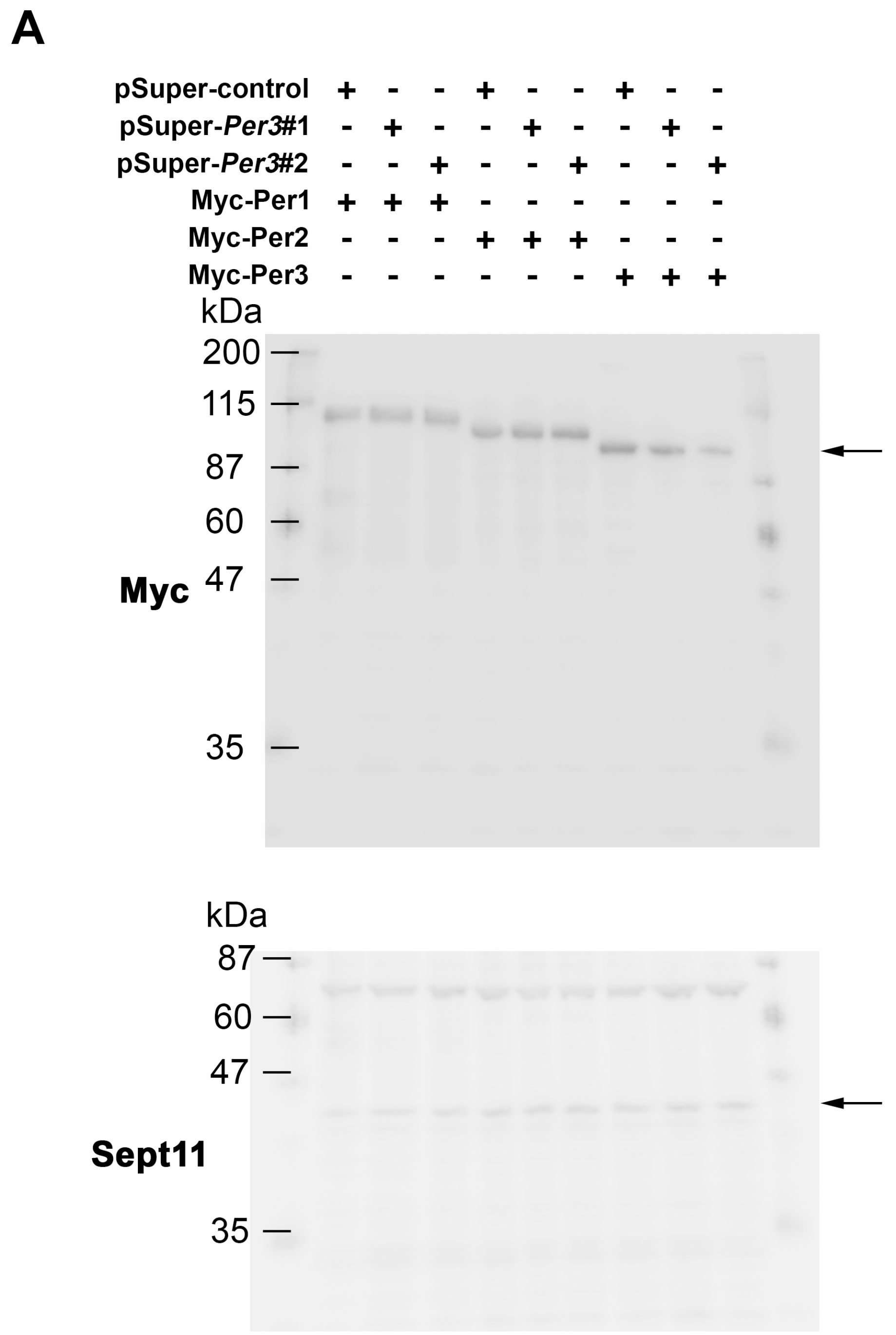

Supplementary Figure 5. Full length blot for densitometry analysis of Figure 1A, as third unrepresented sample Western blot of protein samples immunoblotted with anti-Myc and anti-Sept11. The protein bands used to the analysis in Figure1A are indicated by arrows.

Supplementary video 1. Time-lapse imaging of control neurons migrating in the upper IZ – lower CP.

Supplementary video 2. Time-lapse imaging of migration and morphological change in Per3-deficient neurons in the upper IZ – lower CP.

Supplementary video 3. Time-lapse imaging of control neurons migrating in the CP.

Supplementary video 4. Time-lapse imaging of migration of Per3-deficient neurons in the CP.
